# Machine learning-based prediction of intensive care unit admission in COVID-19 patients presenting with mild respiratory failure

**DOI:** 10.3389/fmed.2026.1724947

**Published:** 2026-02-16

**Authors:** Bahadır Ceylan, Şule Ceylan, Oktay Olmuşçelik, Banu Karaalioğlu, Melih Akan, Meyha Şahin, Mebrule Muğlu, Selda Aydın, Ezgi Yılmaz, Rıdvan Dumlu, Kamil Mert, Abdullah Kansu, Mustafa Düger, Ufuk Süleyman, Esra Demir, İhsan Boyacı, Ali Mert

**Affiliations:** 1 Department of Infectious Diseases and Clinical Microbiology, Medical Faculty, Istanbul Medipol University, Istanbul, Türkiye; 2 Department of Nuclear Medicine, University of Health Science, Gaziosmanpaşa Training ve Research Hospital, Istanbul, Türkiye; 3 Department of Internal Medicine, Medical Faculty, Istanbul Medipol University, Istanbul, Türkiye; 4 Department of Radiology, Medical Faculty, Istanbul Medipol University, Istanbul, Türkiye; 5 Department of Chest Diseases, Medical Faculty, Istanbul Medipol University, Istanbul, Türkiye

**Keywords:** COVID-19, intensive care unit, machine learning, prognosis, steroid

## Abstract

**Introduction:**

Previous studies applying machine learning to predict severe respiratory failure in COVID-19 patients have shown inconsistent results due to variations in study populations and predictor variables. This study aimed to predict intensive care unit admission and identify key predictive factors.

**Methods:**

This retrospective cohort study included patients with COVID-19 who presented with mild respiratory failure, most of whom received oxygen via a mask or nasal cannula. Eight machine learning algorithms—XGBoost, support vector machines, neural networks, k-nearest neighbors, random forest, decision trees, logistic regression, and naïve Bayes—were applied to predict intensive care unit admission.

**Results:**

A total of 392 patients (63.5% male, mean age, 55.0 ± 15.3 years) were included in the study. During follow-up, 80 patients (20.4%) required intensive care unit admission. Among them, 320 (81.6%) received steroid therapy, 301 (76.8%) underwent pulse steroid therapy, and 76 (19%) had been vaccinated. The multilayer perceptron, XGBoost, and radial basis function support vector machine models achieved the best overall performance based on ROC-AUC and accuracy values (ROC-AUC: 0.75, 0.70, and 0.71; accuracy: 0.79, 0.79, and 0.79, respectively). The strongest predictors of intensive care unit admission were low lymphocyte count on the first day, as well as high age, ferritin, body mass index, Charlson Comorbidity Index, and computed tomography score.

**Conclusion:**

Machine learning algorithms can reliably predict intensive care unit admission in COVID-19 patients with mild respiratory failure. These models identified key clinical and laboratory factors that may facilitate early risk stratification and guide treatment planning.

## Introduction

COVID-19 exhibits a broad clinical spectrum, ranging from asymptomatic infection to severe respiratory failure and death (1–22). Numerous studies have applied machine learning (ML) techniques to predict adverse outcomes such as respiratory deterioration, ICU admission, and mortality in COVID-19 patients (1–22). Reported ROC-AUC values in these studies generally range from 0.669 to 0.99, with most exceeding 0.70. In addition, several well-established clinical scoring systems have been developed to predict COVID-19 prognosis (23–27), with ROC-AUC values typically ranging between 0.72 and 0.91, though most cluster between 0.72 and 0.80.

Although these models and scoring systems have demonstrated variable predictive performance, their generalizability remains limited due to substantial heterogeneity in patient populations, disease severity, predictor selection, and sample size. This variability hampers the comparability of findings and limits the ability to draw broadly applicable conclusions. Moreover, most existing studies have included all hospitalized COVID-19 patients regardless of respiratory status and have relied heavily on variables that directly reflect respiratory failure—such as oxygen saturation and respiratory rate. While these variables may enhance predictive accuracy, they may also obscure the contributions of other important clinical and laboratory parameters. Only a few studies have specifically focused on patients already receiving oxygen therapy and managed on general wards (28), leaving a significant gap in understanding disease progression in this clinically important subgroup.

Additional gaps in the literature include selection bias, which may lead retrospective studies to falsely suggest that steroid use is associated with worse outcomes—even though randomized controlled trials have clearly shown that dexamethasone reduces mortality in oxygen-dependent patients (29, 30). Furthermore, although vaccines are known to reduce both the incidence and severity of COVID-19, their impact on disease progression among patients presenting with mild respiratory failure remains poorly understood.

To address these gaps, the present study aimed to use machine learning algorithms to predict ICU admission among patients with COVID-19 presenting with mild respiratory failure and requiring oxygen therapy on admission. A secondary objective was to identify the most influential predictors of clinical deterioration in this patient population, including demographic characteristics, comorbidities, laboratory markers, radiologic findings, vaccination status, and steroid use while minimizing the impact of selection bias.

## Materials and methods

This retrospective cohort study included COVID-19 patients with mild respiratory failure who were admitted to and monitored at Istanbul Medipol University Hospital between January 2020 and January 2022. The study protocol was reviewed and approved by the Institutional Review Board and the Local Ethics Committee of Istanbul Medipol University. The study was conducted in accordance with the ethical standards laid down in the 1964 Declaration of Helsinki and its later amendments, or with comparable ethical standards.

Mild respiratory failure was defined as a respiratory rate of ≥22 breaths per minute at rest, an oxygen saturation level <94% at rest, or a decrease in saturation with minimal exertion. The primary study endpoint was ICU admission. Machine learning algorithms were applied to predict the likelihood of ICU admission. Specific inclusion and exclusion criteria were applied to ensure a well-defined study population.

Inclusion criteria:

- A positive SARS-CoV-2 PCR result from a nasopharyngeal swab obtained within 3 weeks prior to hospital admission.
- Age ≥18 years.
- Hospitalization and treatment for COVID-19 between January 2020 and January 2022.
- Presence of mild respiratory failure.

Exclusion criteria:

- Requirement for high-flow oxygen therapy, continuous positive airway pressure, mechanical ventilation, or extracorporeal membrane oxygenation at initial presentation.
- Admission to the ICU at initial presentation.
- Concomitant conditions unrelated to COVID-19 that could contribute to respiratory failure (e.g., pulmonary edema, pulmonary embolism).
- Absence of respiratory failure at initial presentation.

After applying these exclusion and inclusion criteria, relevant demographic, clinical, and laboratory variables were extracted for analysis.

### Variables evaluated in the study

The following data were collected and analyzed from patient records:

1. *Demographic Information:* Age, sex, and body mass index (BMI).
2. *COVID-19 Vaccination Status:* Only vaccinations administered at least 1 month prior to symptom onset were considered. Patients were categorized according to vaccination status as follows: one dose of inactivated vaccine, two doses of inactivated vaccine, one dose of mRNA vaccine, two doses of mRNA vaccine, or a combination of one dose of mRNA vaccine and one dose of inactivated vaccine.
3. *Comorbidities:* Underlying conditions included liver transplantation, cancer with or without metastasis, heart failure, leukemia, myasthenia gravis, lymphoma, rheumatoid arthritis, aplastic anemia, multiple myeloma, cerebrovascular disease, dementia, Parkinson’s disease, hypertension, ischemic heart disease, diabetes mellitus with or without complications, chronic obstructive pulmonary disease, bronchiectasis, asthma, liver cirrhosis, idiopathic pulmonary fibrosis, chronic renal failure (mild or severe), and renal transplantation. Additional variables included pregnancy, immunosuppression, and prior corticosteroid use. The Charlson Comorbidity Index (CCI) was calculated for each patient (31). Severe renal failure was defined as renal transplantation or a serum creatinine level >3 mg/dL. Patients with renal failure who had not undergone renal transplantation and whose creatinine level was ≤3 mg/dL were categorized as having mild renal failure. Patients with diabetes mellitus were considered to have end-organ damage if they had diabetic retinopathy, nephropathy, or neuropathy.
4. *Laboratory Parameters:* Total neutrophil and lymphocyte counts, as well as serum levels of C-reactive protein (CRP), D-dimer, ferritin, and procalcitonin, measured at the time of initial hospital admission.
5. *Treatments and Interventions Related to COVID-19:* ICU admission and corticosteroid use. Virus-specific antiviral agents were not available in our country during the study period and, therefore, were not administered.
6. *Imaging Findings:* Total and regional lung zone computed tomography (CT) scores were recorded (32). To mitigate the risk of multicollinearity among CT score variables, principal component analysis (PCA) was performed. The first principal component (PC1) accounted for 80% of the total variance and represented a structure in which CT scores from the upper, middle, and lower zones of both lungs contributed similarly in both direction and magnitude. Accordingly, PC1 was interpreted as the composite CT score (overall CT burden) and was used as the representative variable in the analyses.

These variables were subsequently incorporated into statistical analyses to evaluate their associations with ICU admission.

### Statistical analysis

#### General group comparisons

Patients with and without ICU admission during follow-up were compared with respect to the variables described above. Statistical analyses were performed using SPSS software (version 16.0; IBM Corp., Armonk, NY, USA). Categorical variables were expressed as frequencies and percentages. Continuous variables with normal distributions were presented as means ± standard deviations, whereas non-normally distributed variables were summarized as medians (range). Group comparisons were performed using the Student’s t-test for normally distributed continuous variables, as these data met the assumptions of normality, and the Mann–Whitney U test for non-normally distributed continuous variables, which does not assume normality. The chi-square test was used to compare categorical variables. A two-tailed *p*-value <0.05 was considered statistically significant.

To account for potential treatment bias, as pulse steroids were predominantly administered to patients with more severe clinical, laboratory, and radiological findings, comparisons were performed between patients who received pulse steroid therapy and those who did not. Continuous variables with a normal distribution were compared using the Student’s *t*-test, while non-normally distributed continuous variables were analyzed using the Mann–Whitney U test. Categorical variables were compared using the chi-square test. Variables found to be statistically significant in these comparisons were subsequently included in a logistic regression analysis to determine whether an independent difference existed between patients who received pulse steroids and those who did not.

In addition to traditional statistical methods, machine learning approaches were employed to develop predictive models and explore complex relationships among variables.

### Machine learning analysis

**Figure 1:**
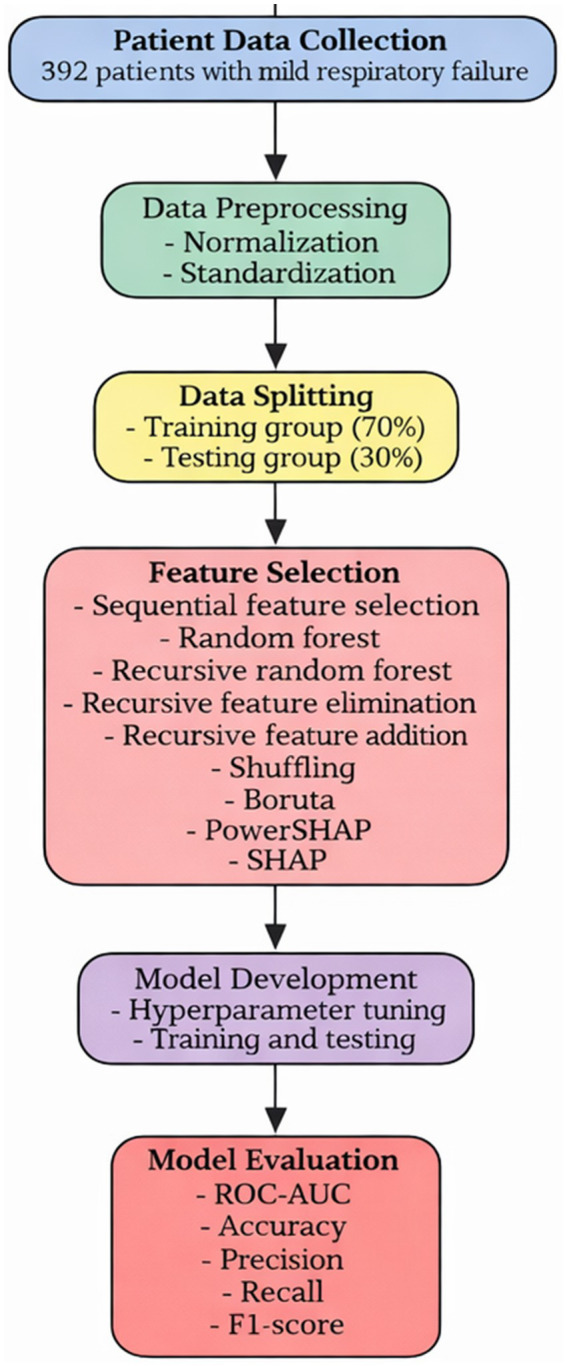
Overview of the machine learning framework for predicting intensive care unit admission in COVID-19 patients.

Several machine learning algorithms were applied to predict ICU admission during follow-up. The overall workflow of the machine learning approach used for predicting ICU admission in COVID-19 patients is illustrated in Figure 1.

#### Data preprocessing

Categorical variables with two categories were encoded using the mapping method, while variables with more than two categories were one-hot encoded. Continuous variables were standardized. Missing values were observed for the following variables: CT scores (37 cases, 9.4%), lymphocyte count (1 case, 0.25%), CRP levels (15 cases, 3.8%), procalcitonin levels (108 cases, 27.5%), ferritin levels (5 cases, 1.2%), symptom duration (45 cases, 11.4%), BMI (15 cases, 3.8%), and onset of dyspnea (4 cases, 1%).

Missing values were imputed using the Iterative Imputer method (sklearn.impute.IterativeImputer), a multivariate regression-based approach that predicts missing values for each variable based on the observed values of other variables through an iterative series of regressions. By default, the Random Forest Regressor was applied.

The dataset was then split into training (70%) and testing (30%) subsets for machine learning analyses.

#### Feature selection

To reduce the number of independent variables and eliminate redundancy, several steps were undertaken. First, constant, quasi-constant, and duplicate variables were removed using the feature-engine package, with thresholds set at 80%, 0.998, and default settings, respectively. Subsequently, multiple feature reduction techniques were evaluated, including:

- Sequential Feature Selection.
- Random Forest (RF).
- Recursive Random Forest.
- Recursive Feature Elimination.
- Recursive Feature Addition.
- Shuffling.
- Boruta.
- SHAP (SHapley Additive exPlanations).
- PowerSHAP.

The reduced feature sets generated by each method were subsequently used as independent variables in the machine learning models developed to predict ICU admission. Among these approaches, we prioritized those that achieved the highest predictive performance (accuracy, ROC-AUC, precision, recall, and F1-score) while using the fewest variables. Based on this assessment, the SHAP-based feature set demonstrated the best balance of accuracy and parsimony; therefore, the predictors identified by SHAP were selected for use in the study. Feature selection was specifically performed using SHAP values derived from a Random Forest model trained on the full set of predictors. Features were ranked according to their mean absolute SHAP values, reflecting their relative contribution to model predictions. Incremental subsets of top-ranked features were evaluated using 5-fold cross-validation, with ROC-AUC as the primary performance metric. The subset of features yielding the highest cross-validated ROC-AUC was selected as the optimal predictor set and used for the development of all subsequent machine learning models. This approach ensured that only the most informative features, as determined by the Random Forest model, were included in the predictive analyses.

Using the SHAP-selected features, machine learning models were constructed to predict ICU admission in COVID-19 patients with mild respiratory failure. Model performance was evaluated using accuracy, ROC-AUC, precision, recall, and F1-score as the primary metrics.

#### Model development, evaluation, and explainability

Machine learning algorithms were implemented in Python to predict ICU admission in COVID-19 patients with mild respiratory failure. Binary classification models were developed using several algorithms, including Extreme Gradient Boosting (XGBoost), linear Support Vector Machine (SVM), radial basis function Support Vector Machine (RBF-SVM), Multilayer Perceptron (MLP), k-Nearest Neighbors (KNN), RF, Decision Tree, Logistic Regression (LR), and Naïve Bayes.

Hyperparameter optimization was performed using GridSearchCV.

Model performance was evaluated using multiple metrics, including accuracy, ROC-AUC, precision, recall, and F1-score. These metrics provided a comprehensive assessment of model performance and guided the selection of the best-performing algorithm.

Given the relatively small sample size and the limited number of ICU admissions, we applied a bootstrapping approach to obtain optimism-corrected estimates of model performance. A total of 500 bootstrap resamples were drawn with replacement from the original dataset. For each resample, the machine learning models were trained and then evaluated on the original dataset to compute performance metrics, including ROC-AUC, accuracy, precision, recall, and F1-score. The optimism-corrected performance for each model was calculated by subtracting the average difference between the performance on the bootstrap samples and that on the original dataset. This procedure provides more robust estimates of model performance and reduces the risk of overfitting, particularly in datasets with a limited number of positive outcomes.

To assess the impact of missing data, particularly for variables with substantial missingness such as procalcitonin (27.5%), a sensitivity analysis was performed. Models were re-trained and evaluated after excluding cases with missing procalcitonin values. Performance metrics, including ROC-AUC, accuracy, precision, recall, and F1-score, were then compared with the results obtained using imputed data. This approach allowed us to evaluate the robustness of our models and the potential influence of imputation on the study findings.

#### Variable importance

The relative importance of variables for predicting ICU admission in COVID-19 patients with mild respiratory failure was assessed using SHAP (SHapley Additive exPlanations) values. This approach enabled a model-agnostic evaluation of each predictor’s contribution, facilitating interpretation and the identification of the most influential clinical, laboratory, and imaging variables. Having established the machine learning framework and selected the most informative features, we next present the results of our analyses in the study cohort.

## Results

We first describe the demographic, clinical, and laboratory characteristics of the patients included in the study.

A total of 392 patients were included in the study, comprising 249 males (63.5%) and 143 females (36.5%), with a mean age of 55 ± 15.3 years. During follow-up, 80 patients (20.4%) were admitted to the ICU, 46 (11.7%) underwent endotracheal intubation, and 47 (12%) died.

Table 1 summarizes the baseline characteristics of patients with and without ICU admission. Among patients requiring ICU care, the CCI, serum CRP, procalcitonin, ferritin, and D-dimer levels, neutrophil counts, age, and the composite CT score (PCA-derived) were higher, whereas lymphocyte counts, the proportion of patients with high body temperature, and the time from symptom onset to dyspnea were lower.

**Table 1:** Comparison of demographic characteristics, clinical features, laboratory parameters, and computed tomography scores between COVID-19 patients with and without intensive care unit admission during hospitalization.

Variables			Patients not admitted to intensive care unit, *n* = 312 (79.6%)	Patients admitted intensive care unit, *n* = 80 (20.4%)	*p*-values
Age (years)			54.85 ± 14.23	55.3 ± 16.69	0.001
Gender	Male		196 (62.8%)	53 (66.3%)	0.570
	Female		116 (37.2%)	27 (33.8%)	
Body Mass Index			28 (17–48)	27 (19–39)	0.386
High body temperature			243 (77.9%)	53 (66.3%)	0.031
Previous COVID-19 vaccination			58 (18.6%)	18 (22.5%)	0.430
Type of COVID-19 vaccine	None		255 (81.7%)	62 (77.5%)	0.549
	One dose inactivated		4 (1.3%)	0 (0%)	
	Two doses inactivated		33 (10.6%)	12 (15%)	
	One dose mRNA		8 (2.6%)	2 (2.5%)	
	Two doses mRNA		7 (2.2%)	1 (1.3%)	
	One dose inactivated + one dose mRNA		5 (1.6%)	3 (3.8%)	
Previous steroid usage			6 (1.9%)	4 (5%)	0.125
Smoking	Never		266 (85.3%)	66 (82.5%)	0.510
	Current		16 (5.1%)	3 (3.8%)	
	Former		30 (9.6%)	11 (13.8%)	
Amount of cigarette smoked (pack-years)			0 (0–40)	0 (0–60)	
Immunosuppression			17 (5.4%)	9 (11.3%)	0.063
Pregnancy			10 (3.2%)	3 (3.8%)	0.733
Comorbidities	Liver transplantation		1 (0.3%)	1 (1.3%)	0.367
	Cancer	Without metastasis	6 (1.9%)	2 (2.5%)	0.668
		With metastasis	8 (2.6%)	2 (2.5%)	1
	Heart failure		9 (2.9%)	5 (6.3%)	0.173
	Leukemia		1 (0.3%)	1 (1.3%)	0.367
	Myasthenia gravis		2 (0.6%)	1 (1.3%)	0.497
	Lymphoma		4 (1.3%)	4 (5%)	0.058
	Rheumatoid arthritis		1 (0.3%)	2 (2.5%)	0.107
	Aplastic anemia		1 (0.3%)	0 (0%)	1.000
	Multiple myeloma		1 (0.3%)	0 (0%)	1.000
	Cerebrovascular diseases		5 (1.6%)	1 (1.3%)	1.000
	Dementia		6 (1.9%)	3 (3.8%)	0.397
	Parkinson’s disease		3 (1%)	1 (1.3%)	1.000
	Hypertension		109 (34.9%)	25 (31.3%)	0.535
	Ischemic heart disease		26 (8.3%)	10 (12.5%)	0.250
	Diabetes mellitus		84 (26.9%)	21 (26.3)	0.903
	Chronic obstructive pulmonary disease		6 (1.9%)	2 (2.5%)	0.668
	Bronchiectasis		1 (0.3%)	0 (0%)	1.000
	Asthma		11 (3.5%)	5 (6.3%)	0.337
	Idiopathic pulmonary fibrosis		1 (0.3%)	2 (2.5%)	0.107
	Chronic renal failure	Mild	8 (2.6%)	3 (3.8%)	0.703
		Severe	7 (2.3%)	3 (3.8%)	0.435
	Renal transplantation		6 (1.9%)	1 (1.3%)	1.000
Charlson Comorbidity Index			1 (0–9)	2 (0–9)	0.001
Time from symptom onset to hospital admission (days)			6 (1–25)	6 (0–30)	0.569
Time from symptom onset to dyspnea onset (days)			7 (1–19)	6 (2–14)	0.030
Steroid use for COVID-19 treatment			253 (81.1%)	67 (83.8%)	0.584
Pulse steroid usage			234 (75%)	67 (83.8%)	0.098
Laboratory values	C-reactive protein (mg/L)		68 (2–404)	120 (14–409)	0.010
	Procalcitonin (ng/mL)		0.11 (0.01–3.82)	0.15 (0.03–8)	0.001
	D-Dimer (ng/mL)		701 (136–9,626)	1,047 (278–9,746)	0.001
	Ferritin (ng/mL)		499 (34–20,598)	765 (59–2,973)	0.002
	Lymphocyte count (/×10^9^/L)		930 (240–6,120)	615 (120–1940)	0.0005
	Neutrophil count (/×10^9^/L)		5,000 (220–24,190)	5,915 (1710–19,850)	0.403
Composite computed tomography score (PCA-derived)			−0.4503 (−3.56 to 8.94)	0.0135 (−3.63 to 10.29)	0.016

Of these patients, 320 (81.6%) received corticosteroid therapy for respiratory failure. The treatment regimen consisted of methylprednisolone administered at 80 mg/day for the first 3 days, 40 mg/day for the subsequent 6 days, and 20 mg/day for the final 3 days. Additionally, 301 patients (76.8%) received 250 mg of pulse steroid therapy for 3 days prior to this regimen.

**Figure 2:**
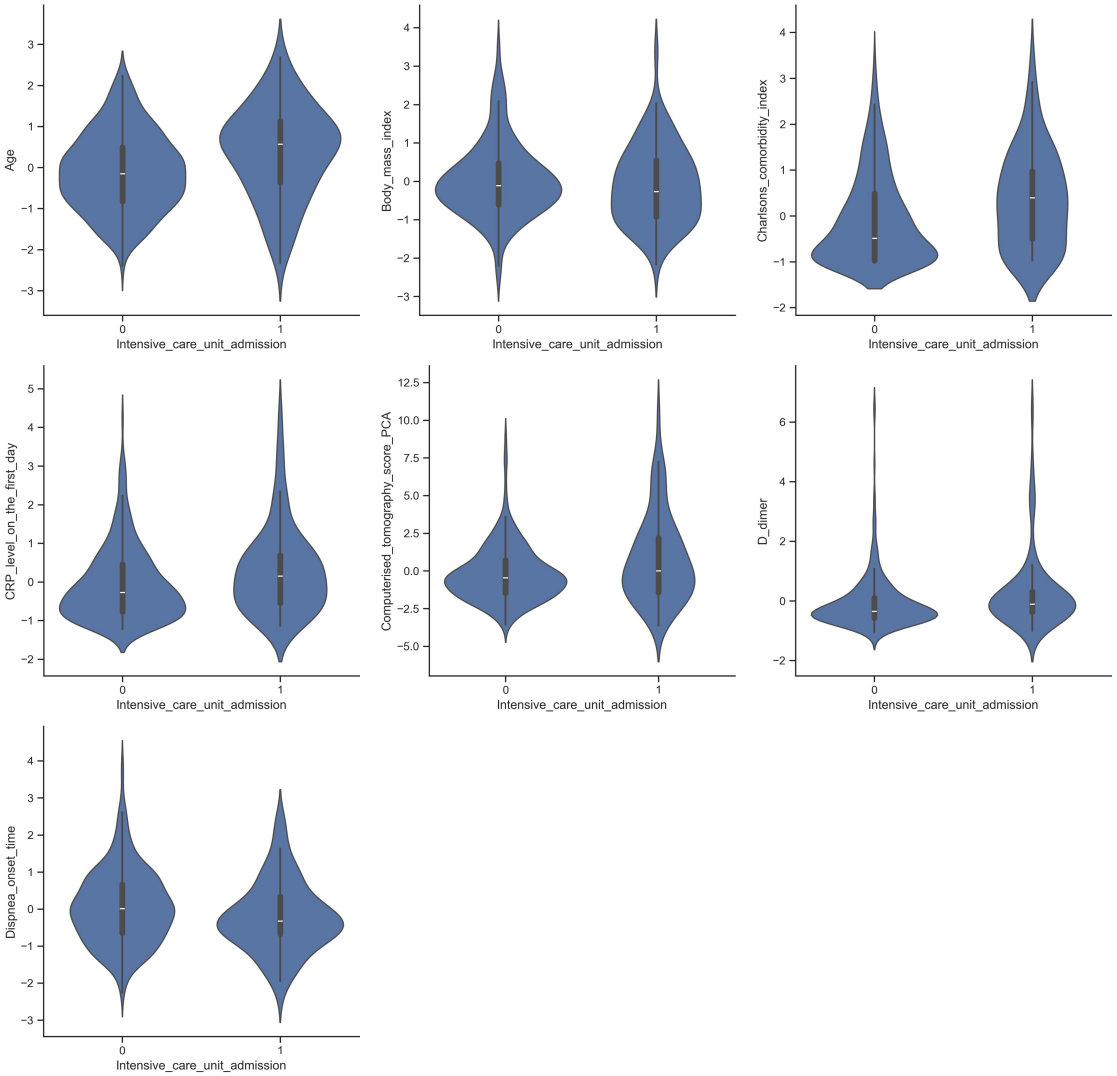
Violin plots illustrating the distribution of selected variables among patients admitted to the intensive care unit (ICU) and those who were not.

**Figure 3:**
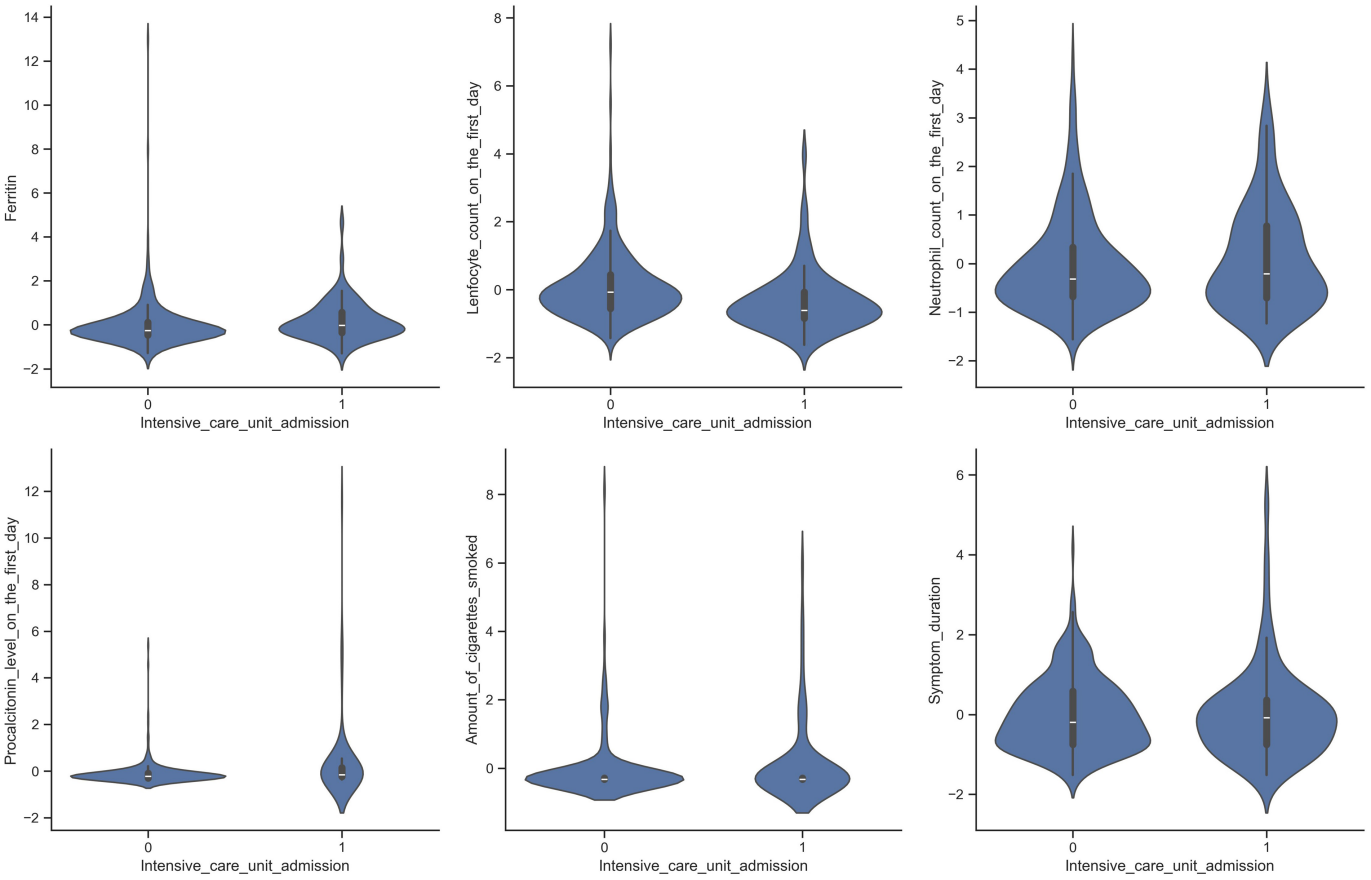
Violin plots illustrating the distribution of selected variables among patients admitted to the intensive care unit (ICU) and those who were not.

Violin plots of the selected variables in patient groups admitted to and not admitted to the ICU are shown in Figures 2, 3.

When comparing patients who received pulse steroid therapy with those who did not, multivariable logistic regression analysis revealed that the pulse steroid group had lower lymphocyte counts, a higher proportion of former smokers compared with never-smokers, and a higher prevalence of fever (Supplementary Table 1). However, no significant differences were observed between the two groups in variables that could potentially influence the decision to administer pulse steroids, including the composite CT score (PCA-derived), serum CRP, procalcitonin, ferritin, D-dimer, neutrophil count, immunosuppression status, and CCI. These findings suggest that clinicians were unlikely to base the decision to initiate pulse steroid therapy solely on lymphocyte count, smoking status, or body temperature, indicating that treatment bias due to pulse steroid use is unlikely.

Building upon these baseline clinical findings, we next performed a comprehensive evaluation of feature selection methods to optimize model performance and assess potential variable interactions.

To determine the most informative feature set, we systematically compared multiple feature selection algorithms, assessing both linear and non-linear interactions among predictors. Sequential Feature Selection substantially reduced model performance, decreasing the ROC-AUC from 0.69 (all features) to 0.61 with the selected subset. LASSO LR did not retain any variables at the tested regularization level, indicating the absence of strong linear predictors under this method. RF provided only a ranking of variable importance without producing a stable subset.

Although RF-based recursive elimination identified five variables that achieved a ROC-AUC of 0.75 internally, performance markedly deteriorated when these features were incorporated into the original machine learning models. Tree-based Recursive Feature Elimination selected 15 variables and yielded a ROC-AUC of 0.57, compared with 0.58 using all features, whereas LR-based Recursive Feature Elimination selected 12 variables and produced identical discrimination (0.60 with selected variables vs. 0.60 with all variables). Permutation-based shuffling using tree models selected six features but showed a decline from ROC-AUC 0.65 (all features) to 0.50 (selected). Similarly, LR-based shuffling selected 11 variables but reduced the ROC-AUC from 0.60 to 0.58 compared with the full-variable model (0.60 with all variables vs. 0.58 with the reduced set).

Recursive feature addition showed limited utility: for tree-based models, the ROC-AUC remained unchanged at 0.58 (0.58 with all variables vs. 0.58 with the reduced set), whereas LR-based addition selected 10 variables and yielded improved performance compared with the full-variable model (0.60 with all variables vs. 0.65 with the reduced set). Boruta identified only two variables, and PowerSHAP selected a small subset as well, but both approaches resulted in poor performance when applied to the original models. All of the above methods produced low evaluation metrics when applied to the original models.

In contrast, the SHAP-selected features consistently maintained or improved model performance, demonstrating superior stability and generalizability. These results suggest that, among the approaches tested to capture potential interactions and non-linear patterns, SHAP-based selection was the most robust and methodologically appropriate strategy for this dataset.

Next, we assessed the predictive performance of the machine learning models in the cohort, considering multiple evaluation metrics.

**Figure 4:**
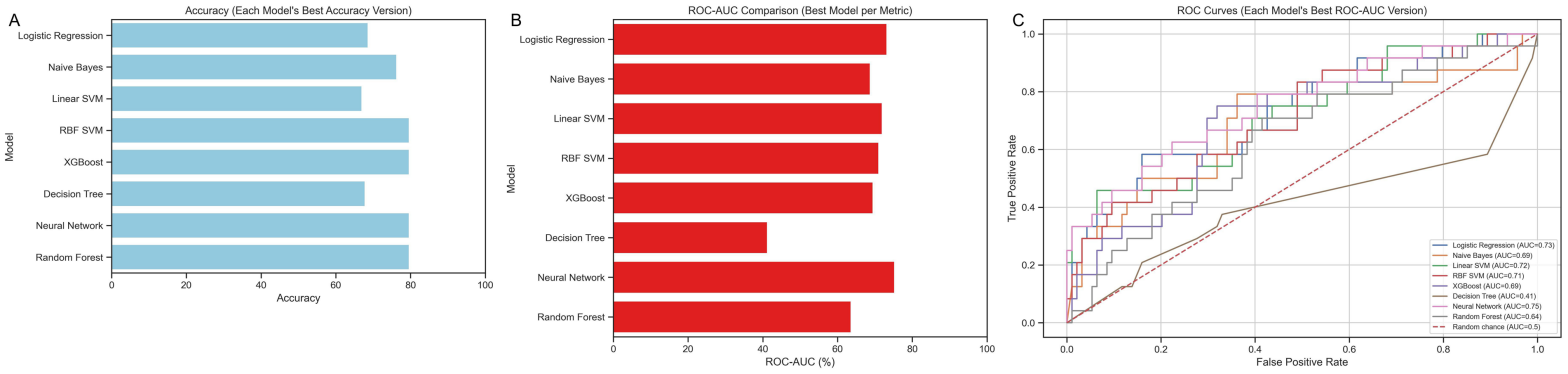
Performance of the machine learning models in predicting intensive care unit admission is shown in panel **(A)** using accuracy. Panel **(B)** shows the area under the ROC curve (ROC-AUC) for each model, and panel **(C)** presents the receiver operating characteristic (ROC) curves of the models.

Several machine learning algorithms were applied to predict ICU admission in COVID-19 patients with mild respiratory failure. Their performances are summarized in Table 2 and illustrated in Figure 4.

**Table 2:** Performance metrics (accuracy, area under the receiver operating characteristic curve [ROC-AUC], precision, recall, and F1-score) and tuning parameters of machine learning algorithms used to predict intensive care unit admission.

Algorithms	Tuned parameters	Accuracy	ROC-AUC	Precision	Recall	F1
Logistic regression	*ROC-AUC*: C = 0.01, maximum iterations = 5,000, penalty = L2, solve r = liblinear *Accuracy:* C = 0.001, maximum iterations = 5,000, penalty = L2, solver = lbfgs	0.68	0.73	0.32	0.58	0.41
Naïve Bayes	–	0.76	0.68	0.42	0.44	0.46
K-Nearest Neighbors	*ROC-AUC*: k = 35 *Accuracy:* k = 12	0.79	0.68	0.10	0.20	0.21
Linear Support Vector Machine	*ROC-AUC*: C = 9 *Accuracy*: C = 2	0.66	0.71	0.31	0.54	0.40
Radial basis function Support Vector Machine	*ROC-AUC*: C = 200, gamma = 0.0001 *Accuracy:* C = 1, gamma = 5	0.79	0.71	0.52	1.00	0.44
Multilayer Perceptron	*ROC-AUC*: Activation = logistic, alpha = 0.1, hidden layer sizes = (5, 3), solver = adam *Accuracy:* Activation = logistic, alpha = 0.1, hidden layer sizes = (5, 3), solver = sgd	0.79	0.75	0.36	1.00	0.47
XGBoost	*ROC-AUC:* Learning rate = 0.02, maximum depth = 4, n_estimators = 100, subsample = 0.6. *Accuracy:* learning rate = 0.02, maximum depth = 5, n_estimators = 100, subsample = 0.6	0.79	0.70	0.36	0.50	0.41
Decision Tree	*ROC-AUC*: Maximum depth = 5, minimum samples split = 39 *Accuracy:* Maximum depth = 8, minimum samples split = 4	0.67	0.41	0.23	0.33	0.25
Random Forest	*ROC-AUC*: Maximum depth = 3, maximum features = 2, minimum samples split = 2, n_estimators = 10 *Accuracy:* Maximum depth = 10, maximum features = 2, minimum samples split = 2, n_estimators = 1,000	0.79	0.63	0.60	0.37	0.33

Considering the ROC-AUC values, all models except KNN, RF, Decision Tree, and Naïve Bayes demonstrated good discriminatory ability, with the MLP and LR achieving the highest ROC-AUC values (0.75 and 0.73, respectively).

The Naïve Bayes, KNN, RBF-SVM, MLP, RF, and XGBoost models achieved favorable accuracies ranging from 0.76 to 0.79. In contrast, LR, Linear SVM, and Decision Tree exhibited relatively lower accuracies (0.68, 0.66, and 0.67, respectively).

When both accuracy and ROC-AUC values were considered together, the RBF-SVM, MLP, and XGBoost models demonstrated the best overall performance (ROC-AUC = 0.71, 0.75, and 0.70; accuracy = 0.79, 0.79, and 0.79, respectively).

Notably, the MLP and RBF-SVM models achieved perfect recall (1.0), indicating an excellent ability to identify all patients requiring intensive care, although with moderate precision. Other models showed comparatively lower precision, recall, and F1-scores.

The bootstrapped performance metrics of all machine learning models are summarized in Supplementary Table 2. In this study of 392 COVID-19 patients, including 80 (20.4%) ICU admissions, we evaluated 15 clinical and laboratory variables using multiple machine learning algorithms. Overall, the models demonstrated moderate predictive performance, with optimism-corrected ROC-AUCs ranging from 0.63 to 0.68 and accuracies ranging from 0.63 to 0.80. These results indicate that the selected variables could be useful for early ICU risk stratification, while acknowledging the limitations imposed by the small sample size and low event rate.

A sensitivity analysis was performed to evaluate the impact of excluding procalcitonin, a variable with substantial missing data (27.5%), on model performance (Supplementary Tables 3, 4). Overall, most models demonstrated largely consistent ROC-AUC and accuracy values, suggesting that imputation did not substantially bias the results. However, some models, particularly tree-based approaches (Decision Tree, RF, XGBoost) and Naïve Bayes, exhibited more pronounced changes in performance metrics.

To better understand the contributions of individual variables to model predictions, we conducted SHAP analysis on the trained models.

**Figure 5:**
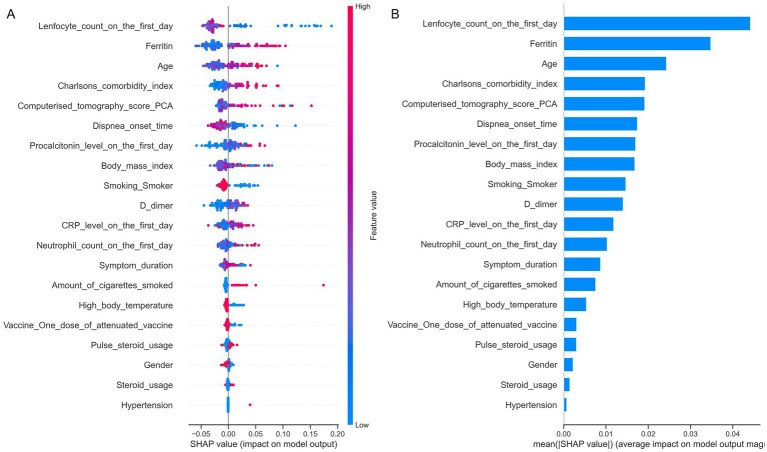
Ranked importance of variables for predicting intensive care unit admission in COVID-19 patients with mild respiratory failure. **(A)** SHAP summary plot showing the impact of each variable on the model output. Each dot represents a single patient. The horizontal position of a dot reflects the effect of that variable on the predicted risk of ICU admission (positive values increase risk, negative values decrease risk). The color of each dot represents the actual feature value for that patient (red = high value, blue = low value). Variables are ranked from top to bottom according to their overall importance in the model. **(B)** Bar plot of mean absolute SHAP values, showing the average contribution of each variable to the model’s predictions, allowing comparison of variable importance across the cohort.

SHAP analysis indicated that the key contributors to the model’s predictions included lower lymphocyte counts, higher neutrophil counts, advanced age, and elevated levels of ferritin, procalcitonin, D-dimer, and CRP. Other important predictors were higher BMI, increased CCI, higher CT scores, delayed hospital admission after symptom onset, shorter time from symptom onset to dyspnea, greater total cigarette consumption, not being a current smoker, and absence of fever (Figure 5).

## Discussion

This study demonstrates that among the evaluated machine learning approaches, the MLP, XGB, and RBF-SVM achieved the highest overall predictive performance, with ROC-AUC values between 0.70 and 0.75 and an accuracy of 0.79. The findings further indicate that the most influential determinants of ICU admission were a low lymphocyte count on the first day, advanced age, elevated ferritin levels, higher BMI, increased CCI, and greater CT severity.

Machine learning-based models can outperform traditional statistical approaches in predicting adverse outcomes in COVID-19, supporting the potential value of ML-driven early risk stratification (33). This evidence also provides the rationale for our use of machine learning methods in the present study.

The highest ROC-AUC was achieved by the MLP model (0.75), likely due to its ability to capture non-linear relationships and interactions between variables (34). Most other models showed similar moderate performance (ROC-AUC 0.70–0.71), while KNN, RF, Naïve Bayes, and Decision Tree performed less well (ROC-AUC 0.68, 0.63, 0.68, and 0.41, respectively). The lower performance of these models may be attributed to limitations in handling complex patterns, feature correlations, or class imbalance, as previously reported (35–38).

The highest accuracy values were achieved by the Naïve Bayes, KNN, RBF-SVM, MLP, XGBoost, and RF models (76%–79%), whereas LR, Linear SVM, and Decision Tree models showed relatively lower values (66%–68%). LR and Linear SVM may have underfit due to their linear decision boundaries, while the limited structure of the Decision Tree likely reduced its accuracy (35, 39, 40).

Non-linear and/or ensemble-based methods, including MLP, XGBoost, and RBF-SVM, demonstrated consistently high and balanced performance in terms of both ROC-AUC and accuracy by effectively capturing complex patterns in the data (34, 41–43). These results suggest that, given the characteristics of the dataset and the sample size, non-linear and ensemble-based approaches may be more suitable for achieving accurate class separation.

Our ML models demonstrated reasonable discrimination for ICU admission, with ROC-AUC and accuracy indicating good apparent performance. Bootstrapped, optimism-corrected estimates were slightly lower, reflecting the limited dataset, but still suggest that selected clinical and laboratory variables can aid early ICU risk stratification, emphasizing the need for validation in larger or external cohorts.

Sensitivity analysis excluding procalcitonin (27.5% missing) showed that most models, including LR and MLP, maintained similar ROC-AUC and accuracy, suggesting minimal bias from imputation. Tree-based models (Decision Tree, RF, XGBoost) and Naïve Bayes were more affected, indicating that missing data impact varies by model type.

Our ML models showed moderate performance (max ROC-AUC 0.75, accuracy 0.79) in a relatively homogeneous cohort with mild respiratory failure, where clinical and laboratory differences between patients who deteriorate and those who do not are subtle, inherently limiting model discrimination. Despite this, the models captured meaningful signals for early risk stratification while complementing clinical judgment. Although broadly comparable to previous studies (ROC-AUC 0.67–0.99), direct comparisons are limited by differences in endpoints, predictors, sample sizes, and patient severity (1–20).

Most prior studies included patients with and without respiratory failure and used direct indicators like oxygen saturation, dyspnea, and respiratory rate, making severe cases easier to identify (1–20). In contrast, our study focused on ward-managed patients with mild respiratory failure, excluding both very severe cases and patients without respiratory failure, and did not include strong prognostic markers. Despite this, our models achieved ROC-AUC and accuracy comparable to previous studies, demonstrating robustness in a more homogeneous population (1–20).

Table 3 summarizes well-established COVID-19 risk scores alongside our model performance (23–27). Overall, our models showed accuracy and ROC-AUC comparable to most clinical scores, except COVID-GRAM and CALL, which performed slightly better. The narrower severity spectrum of our mild respiratory failure cohort likely explains this difference. Nevertheless, using routinely available clinical, laboratory, and radiological data, our models provide useful early risk stratification even in a relatively homogeneous population.

**Table 3:** Comparison of published COVID-19 severity scores and the machine learning models developed in this study for predicting ICU admission and clinical deterioration.

Model/score	ROC-AUC	Accuracy	Outcome/key variables
4C mortality score (39)	0.78 (derivation) / 0.76 (validation)	–	Mortality; age, sex, number of comorbidities, respiratory rate, oxygen saturation, Glasgow Coma Scale, urea, C-reactive protein
COVID-GRAM (40)	0.88 (development)	–	Intensive care unit admission, mechanical ventilation, death; age, X-ray infiltration, hemoptysis, dyspnea, number of comorbidities, cancer history, neutrophil-to-lymphocyte ratio, lactate dehydrogenase, bilirubin
CALL score (41)	0.91	–	Clinical deterioration defined as body temperature or respiratory rate ≥30, oxygen saturation ≤93%, PaO₂/FiO₂ ≤ 300, or need for mechanical ventilation; age, comorbidity count, lymphocyte count, lactate dehydrogenase
SOARS score (42)	0.82 (derivation) / 0.80 (validation) / 0.72 (ISARIC validation)	–	Intensive care unit admission or respiratory deterioration; oxygen saturation, obesity, age, respiratory rate, history of stroke
qCSI score (43)	0.81	0.82	Requirement of more than 10 liters/min of oxygen within the first 24 h of emergency admission; respiratory rate, oxygen saturation, oxygen flow rate
This study (machine learning models)	0.73 for Logistic Regression; 0.75 for Multilayer Perceptron	0.79 for K-Nearest Neighbors, Radial Basis Function Support Vector Classifier, Extreme Gradient Boosting, Random Forest, and Multilayer Perceptron	Intensive care unit admission; lymphocyte counts, neutrophil counts, advanced age, ferritin, procalcitonin, D-dimer, C-reactive protein, body mass index, Charlson Comorbidity Index, computerized tomography scores, time from symptom onset to hospital admission, time from symptom onset to dyspnea, total cigarette consumption, smoking status, high body fever

SHAP analysis identified the strongest predictors of poor prognosis in our cohort, with low lymphocyte count emerging as the most important. Lymphopenia in COVID-19 results from mechanisms such as TNF-*α*–mediated apoptosis, increased peripheral consumption, and ACE2- or CD147-related cytopathic effects (44–47). Elevated neutrophils may further contribute through cytotoxic activity (47, 48). Consistent with prior studies, our findings showed that lower lymphocyte counts at admission were strongly associated with progression to severe respiratory failure (48, 49).

Literature shows that COVID-19 patients with higher CT scores are more likely to develop severe disease and poor outcomes, including death, mechanical ventilation, and ICU admission (21, 22, 50–54). Our findings align with these reports.

Excessive activation of the innate immune system and cytokine release—particularly IL-6—play a central role in severe COVID-19 (55, 56). This hyperinflammatory state drives neutrophilia, promotes hypercoagulability reflected by elevated D-dimer, and induces macrophage activation that markedly increases ferritin levels (56–62). CRP likewise rises through IL-6–mediated acute-phase signaling (55, 63). Consistent with this shared pathophysiological pathway, our study found that higher neutrophil counts, D-dimer, ferritin, and CRP levels at admission were all associated with worse clinical outcomes, including respiratory deterioration and increased need for intensive care.

Consistent with the literature, elevated procalcitonin, a marker of bacterial infection and tissue hypoxia, was associated with a higher risk of respiratory deterioration at admission in our cohort (64, 65).

Previous studies have shown that higher Charlson Comorbidity Index (CCI) scores, advanced age, and increased body mass index (BMI) are associated with greater COVID-19 severity (66–68), and our findings were consistent with these observations.

Another important predictor identified was the short interval between symptom onset and the development of dyspnea, indicating that earlier onset of dyspnea substantially increases the risk of adverse outcomes. To our knowledge, this association has not been previously reported, representing a novel finding of our study.

Previous studies show that COVID-19 patients who smoke are more likely to require intensive care (69). Smoking induces structural lung changes and alters immune responses, increasing infection severity and mortality (70, 71). In our cohort, current smokers appeared less likely to be admitted to the ICU; however, this is likely due to the very small number of current smokers (*n* = 19). Notably, higher cumulative cigarette exposure among current and former smokers was associated with increased ICU admission, consistent with prior reports.

Previous studies have shown that corticosteroids reduce mortality in patients with COVID-19, although they do not appear to decrease the need for ICU admission (29, 72). In line with these findings, corticosteroid use in our study was not associated with ICU admission.

Previous studies have shown that mRNA vaccination reduces the risk of severe respiratory failure and COVID-19-related mortality (73–77). In our study, vaccination was not significantly associated with ICU admission, likely reflecting the characteristics of our cohort, which included only patients already presenting with respiratory failure—a stage beyond the primary protective effect of vaccination.

Our study has several limitations. It was retrospective and single-center, which may limit generalizability, though our Istanbul-based hospital serves a diverse population. The sample size was relatively small (392 patients, 80 ICU admissions), yielding an events-per-variable ratio of approximately 5, below the recommended threshold for robust modeling; bootstrapping was used to assess model stability. Despite these limitations, the use of machine learning provides an objective, data-driven approach to early ICU risk prediction. Larger, multicenter studies are needed to validate our findings and further clarify factors influencing severe respiratory failure in COVID-19.

## Conclusion

Machine learning algorithms effectively predicted the risk of ICU admission in COVID-19 patients presenting with mild respiratory failure. The most influential predictors of adverse outcomes were low lymphocyte counts, advanced age, elevated ferritin levels, high CCI, and increased CT scores.

## Data Availability

The raw data supporting the conclusions of this article will be made available by the authors, without undue reservation.
