# Supplementary material for: Machine learning-based prediction of intensive care unit admission in COVID-19 patients presenting with mild respiratory failure
Source: Front Med (Lausanne). 2026 Feb 16;13:1724947. doi: 10.3389/fmed.2026.1724947 (PMC12951780; doi:10.3389/fmed.2026.1724947)
Supplement: Supplementary file 1 [file Table_1.DOCX]

**Supplementary** **Table 1** Multivariable logistic regression analysis comparing patients who received pulse steroid therapy versus those who did not.

| **Variable** | **Odds ratio (OR)** | **95% Confidence Interval (CI)** | **p-value** |
| --- | --- | --- | --- |
| **Lymphocyte count** | 0.999 | 0.999-1 | 0.005 |
| **Ex-smoker (vs. never-smoker)** | 6.659 | 1.497-29.622 | 0.013 |
| **High body temperature** | 2.163 | 1.217-3.845 | 0.009 |
