# Supplementary material for: Machine learning-based prediction of intensive care unit admission in COVID-19 patients presenting with mild respiratory failure
Source: Front Med (Lausanne). 2026 Feb 16;13:1724947. doi: 10.3389/fmed.2026.1724947 (PMC12951780; doi:10.3389/fmed.2026.1724947)
Supplement: Supplementary file 2 [file Table_2.DOCX]

**Supplementary Table 2** Bootstrapped performance metrics of machine learning models for predicting intensive care unit admission in COVID-19 patients (n=392)

| **Model** | **Metric** | **Apparent** | **Optimism-Corrected** | **95% CI Lower** | **95% CI Upper** |
| --- | --- | --- | --- | --- | --- |
| **Logistic Reg.** | **ROC-AUC** | 0.7438 | 0.6835 | 0.6495 | 0.7498 |
|  | **Average Precision** | 0.4004 | 0.3879 | 0.3277 | 0.5679 |
|  | **Accuracy** | 0.7153 | 0.6937 | 0.6610 | 0.7458 |
|  | **F1-score** | 0.4940 | 0.4336 | 0.3830 | 0.4795 |
|  | **Precision** | 0.3727 | 0.3318 | 0.2857 | 0.3810 |
|  | **Recall** | 0.7321 | 0.6276 | 0.5417 | 0.6667 |
| **Naïve Bayes** | **ROC-AUC** | 0.7112 | 0.6179 | 0.4356 | 0.7620 |
|  | **Average Precision** | 0.4047 | 0.3088 | 0.2248 | 0.5088 |
|  | **Accuracy** | 0.7445 | 0.7348 | 0.5763 | 0.8051 |
|  | **F1-score** | 0.4697 | 0.3961 | 0.2596 | 0.5372 |
|  | **Precision** | 0.4079 | 0.3666 | 0.1933 | 0.5455 |
|  | **Recall** | 0.5536 | 0.4151 | 0.2917 | 0.6250 |
| **KNN** | **ROC-AUC** | 0.7369 | 0.6341 | 0.5848 | 0.7235 |
|  | **Average Precision** | 0.3668 | 0.3031 | 0.2600 | 0.4773 |
|  | **Accuracy** | 0.7920 | 0.7769 | 0.7583 | 0.8220 |
|  | **F1-score** | 1.0000 | 0.2092 | 0.0941 | 0.3333 |
|  | **Precision** | 0.0000 | -0.3334 | 0.0000 | 1.0000 |
|  | **Recall** | 1.0000 | 0.1965 | 0.0833 | 0.3135 |
| **Linear SVC** | **ROC-AUC** | 0.7615 | 0.6509 | 0.5217 | 0.7728 |
|  | **Average Precision** | 0.4060 | 0.3788 | 0.2635 | 0.5811 |
|  | **Accuracy** | 0.7117 | 0.6312 | 0.5847 | 0.7288 |
|  | **F1-score** | 0.5153 | 0.3694 | 0.2985 | 0.5077 |
|  | **Precision** | 0.3925 | 0.2771 | 0.2051 | 0.4000 |
|  | **Recall** | 0.7500 | 0.5433 | 0.3750 | 0.7083 |
| **RBF SVC** | **ROC-AUC** | 0.7581 | 0.6669 | 0.5950 | 0.7567 |
|  | **Average Precision** | 0.4003 | 0.3747 | 0.3153 | 0.5735 |
|  | **Accuracy** | 1.0000 | 0.7966 | 0.7966 | 0.7966 |
|  | **F1-score** | 0.5032 | 0.4191 | 0.3448 | 0.4925 |
|  | **Precision** | 0.4286 | 0.4498 | 0.0000 | 0.7894 |
|  | **Recall** | 1.0000 | 1.0000 | 0.0000 | 1.0000 |
| **MLP Classifier** | **ROC-AUC** | 0.7602 | 0.6538 | 0.5931 | 0.7737 |
|  | **Average Precision** | 0.4057 | 0.3775 | 0.2980 | 0.6034 |
|  | **Accuracy** | 0.7956 | 0.7966 | 0.7966 | 0.7966 |
|  | **F1-score** | 0.5093 | 0.3376 | 0.2034 | 0.5000 |
|  | **Precision** | 0.5444 | 0.1587 | 0.1371 | 0.4667 |
|  | **Recall** | 1.0000 | 1.0000 | 1.0000 | 1.0000 |
| **XGBoost** | **ROC-AUC** | 0.9857 | 0.6801 | 0.6041 | 0.7660 |
|  | **Average Precision** | 0.8952 | 0.3321 | 0.3100 | 0.5165 |
|  | **Accuracy** | 0.9635 | 0.7487 | 0.7119 | 0.8220 |
|  | **F1-score** | 0.6818 | 0.3834 | 0.3063 | 0.5306 |
|  | **Precision** | 0.8116 | 0.2865 | 0.2667 | 0.5859 |
|  | **Recall** | 0.8214 | 0.3855 | 0.2917 | 0.6667 |
| **CART** | **ROC-AUC** | 0.8312 | 0.5220 | 0.4681 | 0.7309 |
|  | **Average Precision** | 0.6744 | 0.1263 | 0.1987 | 0.3805 |
|  | **Accuracy** | 0.9124 | 0.6392 | 0.5932 | 0.7712 |
|  | **F1-score** | 0.6122 | 0.2668 | 0.2097 | 0.4837 |
|  | **Precision** | 0.7000 | 0.0950 | 0.1454 | 0.4443 |
|  | **Recall** | 0.8036 | 0.3588 | 0.2500 | 0.7500 |
| **Random Forest** | **ROC-AUC** | 0.8575 | 0.6084 | 0.5741 | 0.7593 |
|  | **Average Precision** | 0.6198 | 0.2013 | 0.2711 | 0.5116 |
|  | **Accuracy** | 1.0000 | 0.8029 | 0.7881 | 0.8220 |
|  | **F1-score** | 0.5106 | 0.3204 | 0.2500 | 0.4923 |
|  | **Precision** | 0.8889 | 0.4575 | 0.3333 | 0.8000 |
|  | **Recall** | 0.6429 | 0.3985 | 0.2917 | 0.6667 |
