# Supplementary material for: Machine learning-based prediction of intensive care unit admission in COVID-19 patients presenting with mild respiratory failure
Source: Front Med (Lausanne). 2026 Feb 16;13:1724947. doi: 10.3389/fmed.2026.1724947 (PMC12951780; doi:10.3389/fmed.2026.1724947)
Supplement: Supplementary file 3 [file Table_3.DOCX]

**Supplementary Table 3** Comparison of machine learning model performance with and without procalcitonin, including ROC-AUC and accuracy.

| **Model** | **ROC-AUC (imputed)** | **ROC-AUC (no procalcitonin)** | **Δ*ROC-AUC** | **Accuracy (imputed)** | **Accuracy (no procalcitonin)** | **Δ*Accuracy** |
| --- | --- | --- | --- | --- | --- | --- |
| Logistic Regression | 0.73 | 0.737 | +0.007 | 0.68 | 0.733 | +0.053 |
| Naïve Bayes | 0.68 | 0.606 | -0.074 | 0.76 | 0.698 | -0.062 |
| KNN | 0.68 | 0.657 | -0.023 | 0.79 | 0.814 | +0.024 |
| Linear SVM | 0.71 | 0.669 | -0.041 | 0.66 | 0.639 | -0.021 |
| RBF SVM | 0.71 | 0.707 | -0.003 | 0.79 | 0.698 | -0.092 |
| MLP | 0.75 | 0.707 | -0.043 | 0.79 | 0.826 | +0.036 |
| XGBoost | 0.70 | 0.649 | -0.051 | 0.79 | 0.651 | -0.139 |
| Decision Tree | 0.41 | 0.618 | +0.208 | 0.67 | 0.500 | -0.17 |
| Random Forest | 0.63 | 0.732 | +0.102 | 0.79 | 0.744 | -0.046 |

*Δ indicates the change in performance metric after excluding procalcitonin, calculated as (no procalcitonin) − (imputed).
