# Supplementary material for: Machine learning-based prediction of intensive care unit admission in COVID-19 patients presenting with mild respiratory failure
Source: Front Med (Lausanne). 2026 Feb 16;13:1724947. doi: 10.3389/fmed.2026.1724947 (PMC12951780; doi:10.3389/fmed.2026.1724947)
Supplement: Supplementary file 4 [file Table_4.DOCX]

**Supplementary Table 4** Comparison of machine learning model performance with and without procalcitonin, including precision, recall, and F1-score.

| Model | **Precision (imputed)** | **Precision (no procalcitonin)** | **Δ*Precision** | **Recall (imputed)** | **Recall (no procalcitonin)** | **Δ*Recall** | **F1-score (imputed)** | **F1-score (no procalcitonin)** | **Δ*F1** |
| --- | --- | --- | --- | --- | --- | --- | --- | --- | --- |
| Logistic Regression | 0.32 | 0.357 | +0.037 | 0.58 | 0.667 | +0.087 | 0.41 | 0.465 | +0.055 |
| Naïve Bayes | 0.42 | 0.238 | -0.182 | 0.44 | 0.333 | -0.107 | 0.46 | 0.278 | -0.182 |
| KNN | 0.10 | 0.117 | +0.017 | 0.20 | 0.267 | +0.067 | 0.21 | 0.266 | +0.056 |
| Linear SVM | 0.31 | 0.265 | -0.045 | 0.54 | 0.6 | +0.06 | 0.40 | 0.367 | -0.033 |
| RBF SVM | 0.52 | 0.323 | -0.197 | 1.00 | 0.667 | -0.333 | 0.44 | 0.392 | -0.048 |
| MLP | 0.36 | 0.551 | +0.191 | 1.00 | 1.00 | 0.00 | 0.47 | 0.60 | +0.13 |
| XGBoost | 0.36 | 0.296 | -0.064 | 0.50 | 0.733 | +0.233 | 0.41 | 0.434 | +0.024 |
| Decision Tree | 0.23 | 0.231 | +0.001 | 0.33 | 0.618 | +0.288 | 0.25 | 0.358 | +0.108 |
| Random Forest | 0.60 | 0.348 | -0.252 | 0.37 | 0.762 | +0.392 | 0.33 | 0.421 | +0.091 |

*Δ indicates the change in performance metric after excluding procalcitonin, calculated as (no procalcitonin) − (imputed).
